# Increased glucocorticoid receptor activity and proliferation in metastatic colon cancer

**DOI:** 10.1038/s41598-019-47696-2

**Published:** 2019-08-02

**Authors:** Dan Tian, Miao Tian, Gang Han, Jin-Long Li

**Affiliations:** 1 Department of Anesthesiology, The Second Hospital of Jilin University, Changchun, China; 2 Department of Gynecology, The Second Hospital of Jilin University, 218 Ziqiang Street, Changchun, 130041 China; 3 Department of Gastrointestinal Surgery, The Second Hospital of Jilin University, Changchun, China

**Keywords:** Colon cancer, Colon cancer

## Abstract

Metastasis is regarded as the fatal hallmark for colon cancer, but molecular mechanisms responsible for it have remained poorly defined. Glucocorticoid receptor (GR) within the tumor microenvironment mediates the effects of stress hormones which are used in clinics for their inflammation-modulatory and immunosuppressive properties. Further, epigenetic activation of GR promotes tumor heterogeneity and metastasis. Here, we sought to investigate the correlation between GR activation and proliferation and invasion in metastatic colon cancer microenvironment. We used proliferation/invasion assays, western blot, RT-qPCR, immunofluorescence staining and quantitative methylation to study glucocorticoid-GR signaling, including the involvement of CDK1, in human colon adenocarcinoma cell lines HT29 and T84 (a representative metastatic cell line). Nuclear expression levels of GR were significantly upregulated in metastatic T84 cells, and glucocorticoid derivative, dexamethasone (DEX) treatment caused increased proliferation and invasion in T84 cell, compared to HT29 cell. DEX treatment induced CDK1 expression which was accompanied by reduced CDK1 methylation, indicating epigenetic regulation. Depletion of GR suppressed proliferation of metastatic colon carcinoma cells and depletion of CDK1 had similar suppressing effects on proliferation as well as invasion of metastatic cells. Our study suggests that glucocorticoid-GR-CDK1 signaling induces proliferation and invasion of colon cancer cells and therapies involving the use of glucocorticoids need to exercise caution and re-evaluation.

## Introduction

Colorectal cancer (CRC) is one major cancer that results in numerous cancer-related deaths around the world^1,2^. The 5-year overall survival of CRC patients differs for patients representing different clinical stages. While the 5-year survival for stage I CRC patients is 93%, it stands at an unacceptable rate of 8% for stage IV patients^3^. At present, surgical resection is considered the most effective treatment for CRC, with only a modest benefit afforded by chemotherapy^4^. Despite all the research, CRC survival rates have not improved over past several decades. Therefore, discovery and validation of clinically relevant diagnostic and prognostic markers is urgently needed.

Liver is a frequent and often the first metastatic site for advanced CRC. It is also the exclusive site of metastatic spread in 30–40% of advanced CRC patients. Hematogenous spread of CRC often follows a pattern wherein hepatic metastasis occurs first, followed by the involvement of portal vein in intra-hepatic spread, leading ultimately to systemic circulation, thus, resulting in surgical resection of hepatic metastatic modules as an effective approach to counter metastatic spread and improve overall survival of patients^5^. As would be expected, metastatic CRCs are particularly lethal, with a median survival of eight months when left untreated^6^. However, CRC patients, who have undergone surgery for the resection of hepatic metastases, report much better prognosis, provided that the metastatic spread is not substantially more exhaustive^7^.

Our primary hypothesis for this study was that glucocorticoid-GR signaling promotes cell proliferation in metastatic CRC. We show that treatment of metastatic human colon carcinoma cell line, T84 with dexamethasone (DEX), a potent synthetic glucocorticoid, which is reminiscent of signaling within the tumor microenvironment, led to enhanced proliferation as well as invasion, compared to non-metastatic CRC cell line, HT29. We further show that DEX promoted cell proliferation in T84 by inducing expression of cyclin-dependent kinase 1 (CDK1) expression. CDKs consist of a family of protein kinases driving the major events of cell cycle in eukaryotic cells^8^, and it has been reported that de-regulated CDKs not only induce rapid tumor growth but also support proliferation of cancer cells^9,10^. In view of our findings, we advocate due caution while including glucocorticoids in the treatment strategy for CRC patients, particularly the CRC patients with metastatic and advanced disease.

## Materialas and Methods

### Cell culture

HT29 and T84, the human colon carcinoma cell lines, were obtained from ATCC, USA, and cultured in RPMI-1640 medium supplemented with 10% fetal bovine serum. The cells were grown in a humidified incubator at 37 °C and 5% CO_2_.

### Reagents

DEX was from Sigma-Aldrich (Shanghai, China). CDK1, β-actin, p65, and GR antibodies were from Santa Cruz Biotechnology (TX, USA). siRNAs were designed and synthesized by IDT DNA (USA).

### Western blot

We used nuclear extraction kit (Millipore, China) to prepare the nuclear and cytosolic cell extracts. 1× SDS loading buffer was added to the proteins, and SDS electrophoresis was performed by resolving samples on SDS–10% PAGE gels. This was followed by transfer onto PVDF membranes (Bio-Rad, China) and blocking of membranes using 5% nonfat milk powder in PBST, and finally overnight incubation with primary antibodies at 4 °C and an hour long incubation with appropriate secondary antibodies with extensive washings in between. We used β-actin as loading control. Immun-Star HRP Substrate (Bio-Rad, China) was used for the visualization of protein bands on autoradiograms.

### Immunofluorescence (IF) staining

Cells were fixed using 4% paraformaldehyde in PBS, and permeabilized for 10 minutes, using 0.5% Triton X-100 in PBS. This was followed by washing and overnight incubation at 4 °C with appropriate antibodies; washing to remove excess antodies and incubation with fluorophore-conjugated secondary antibody (Alexa Fluor 488 goat anti-rabbit IgG) for 1 hour at room temperature. Finally, cells were counterstained with 4′,6-diamidino-2-phenylindole DAPI (Invitrogen, China) and visualized using a fluorescence microscope (Olympus, Japan).

### Preparation of total RNAs

Total RNA content of cells was extracted by directly adding Trizol-LS reagent (Invitrogen, China) to the plates and following manufacturer’s protocol. We collected aqueous phase after the addition of chloroform and added ethanol, followed by centrifugation to precipitate RNAs out. RNA pellet was dissolved in 10 μL of nuclease-free water and the purity and concentration measured on a NanoDrop instrument (ThermoScientific, China).

### Quantitative RT-PCR

For quantitative estimation of mRNAs, we used oligo-dT primers for total cDNA synthesis, using the Superscript II Reverse Transcription kit (Invitrogen, China), and used ABI7300 machine (Applied Biosystems, China). mRNA levels were determined with relative to GAPDH expression using the SYBR Green PCR kit (Qiagen, China), respectively. Fold change was determined by the method of cycle threshold (CT) in the formula of 2^−ΔΔCT^. GAPDH forward, 5′-CTCCCGCTTCGCTCTCTG-3′, GAPDH reverse, 5′- CTGGCGACGCAAAAGAAG -3′; CDK1 forward, 5′- AAAGCCTAGGGCAGAGTGGT-3′, CDK1 reverse, 5′- GGTTTGGTAGAACTGGTGCAA-3′.

### Chromatin Immunoprecipitation (ChIP)

ChIP was performed, exactly as described previously^11^. Fold enrichment was calculated with the method of cycle threshold in the formula of 2^−ΔΔCT^. The primers (forward/reverse) used for the amplicon to *CDK1* promoter were 5′- AAAGAAGAACGGAGCGAACA-3′/5′- GCTAGAGCGCGAAAGAAAGA-3′. Control primers (forward/reverse) for a non-binding site (a human DNA alpha satellite) were 5′-TCTCAGAATCTTCCTTTTGATGTG-3′/5′-CCAGTTGCAGATCCTACAAAGA-3′.

### Methylation quantitation

We determined the levels of methylation in colon carcinoma cells using FFPE Bisulfite conversion kit from Active Motif (Shanghai, China). Manufacturer’s protocol was used without any modifications and primers were designed, using established methodology^12^.

### Cell proliferation assay

MTT [3-(4, 5-dimethylthiazol-2-yl)-2, 5-diphenyltetrazoliumbr-omide] - based assay was performed to study cell proliferation, as previously described^13,14^. Cells were seeded into 96-well plates (5,000 cells/well in 200 µL medium) for 24 hr prior to the assay. Once the assay was complete, 20 µL of 5 mg/ml MTT (Sigma, China) solution was added to every single well and the plates incubated for another 4 hr in the cell culture incubator. This was followed by replacement of liquid with 150 µL DMSO (Sigma-, China). Finally, OD was determined at 490 nm using multi-microplate test system (Victor3, MA, USA).

### Cell invasion assay

Cell invasion was studied using cell invasion kit purchased from R&D Systems (Shanghai, China) which contained 96-well boyden chamber with a polyethylene terephthalate membrane pre-coated with Basement Membrane Extract (BME) at medium density, to investigate cell invasion *in vitro*. Treated or control cells were placed in the top chamber and those that invaded through the BME and filter pores to the bottom of filter were quantified using a cell dissociation / calcein AM solution.

### Statistical analysis

Data is expressed as mean ± standard deviation (SD). We used one-way ANOVA and Student’s *t*-test to compare three and two groups, respectively. p < 0.05 was considered significantly different.

## Results

### GR is constitutively activated in metastatic colon carcinoma cell line

**Figure 1:**
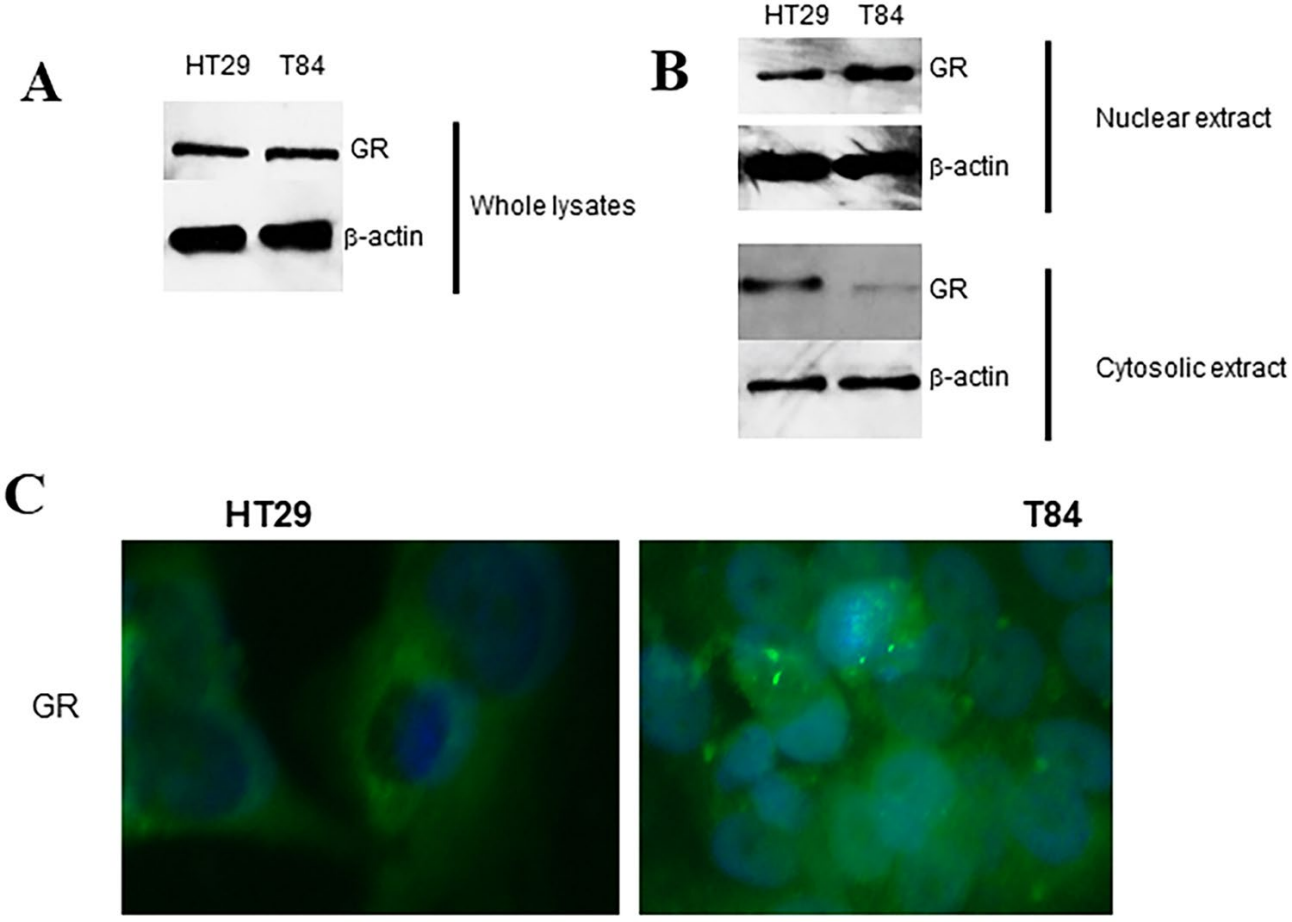
GR expression in HT29 and T84 colon carcinoma cell lines. (**A**) Representative image from western analysis of whole cell lysates from HT29 and T84 colon carcinoma cell lines. (**B**) Representative image from western analysis of nuclear and cytosolic extracts of HT29 and T84 colon carcinoma cell lines. (**C**) Immunofluorescence staining was performed for a direct visualization of cellular distribution of GR in HT29 and T84 cells.

The transcription factor, GR, is expressed in almost all types of cells within the human body. Upon ligand glucocorticoid binding, the glucocorticoid-GR (hormone-receptor) complex translocates to nucleus and then acts on target gene promoter resulting in modulation of gene transcription. In order to determine whether GR is activated in colon cancer, we performed western blotting analysis on extracts (nuclear and cytosolic) prepared from HT29 or T84 cell. As shown in Fig. 1A, we did not notice any difference in GR in protein abundance between these cell lines. However, GR levels were remarkably increased in the nuclear extracts in T84 cells, compared to HT29 cells (Fig. 1B). Moreover, the increased nuclear localization in T84 cells was also visualized by immunofluorescence staining (Fig. 1C). Taken together, these results suggest that GR is constitutively activated in metastatic colon carcinoma cells at a higher rate than the non-metastatic colon carcinoma cells.

### Glucocorticoid-GR signaling promotes metastatic colon carcinoma proliferation

**Figure 2:**
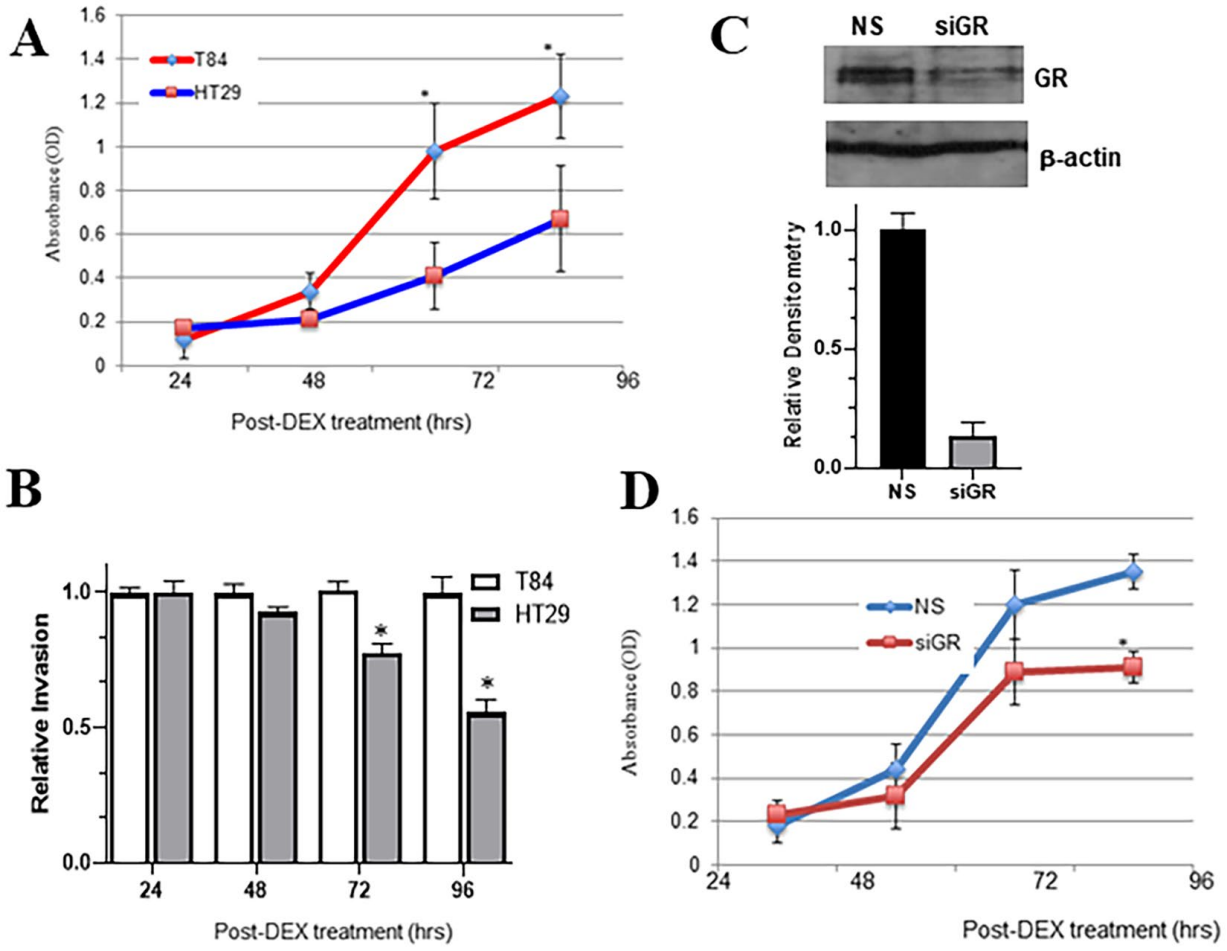
DEX treatment results in increased proliferation of metastatic T84 colon carcinoma cells. Colon adenocarcinoma cell lines HT29 or T84 were exposed to 1 μM DEX and the proliferation of colon carcinoma was analyzed by (**A**) MTT proliferation (n = 3 individual experiments) and (**B**) invasion assay. For the invasion assay, the fluorescence of T84 cells was set as 1, and relative fluorescence of HT29 cells is reported. *p < 0.01. (**C**) Representative image from western analysis of whole cell lysates from T84 colon carcinoma cell line transfected with non-specific (NS) or GR siRNA (siGR) for 48 hr. Densitometric analysis of GR band from the representative western band was done, respective to the control β-actin band, with the value of GR band under NS conditions set as 1. (**D**) After 48 hr of siRNA transfection, the proliferation of colon carcinoma cells T84 was analyzed by MTT proliferation assay (n = 3 individual experiments) following DEX treatment. *p < 0.01.

HT29 or the metastatic cells T84 were exposed to DEX (1 μM) for 24 hr or the DMSO (control). MTT proliferation assay was then performed and the results show that DEX-treatment caused a significant increase in cancer cell proliferation in T84 cells, compared to HT29 cells (Fig. 2A). Then, we performed invasion assay and observed that, similar to proliferation, DEX-treatment resulted in more invasion of T84 cells, compared to HT29 cells (Fig. 2B). To verify that these observed results were an outcome of glucocorticoid-GR interaction, we silenced GR in T84 cells, using siRNA against GR. The silencing was very efficient (Fig. 2C) and the silencing of GR, followed by DEX treatment, resulted in much reduced proliferation (Fig. 2D) verifying an important role of GR in proliferation of metastatic cells T84. These results suggested that glucocorticoids stimulate metastatic colon cancer cell proliferation by activating GR.

### Glucocorticoid-GR signaling induces gene expression of CDK1 in metastatic colon carcinoma

**Figure 3:**
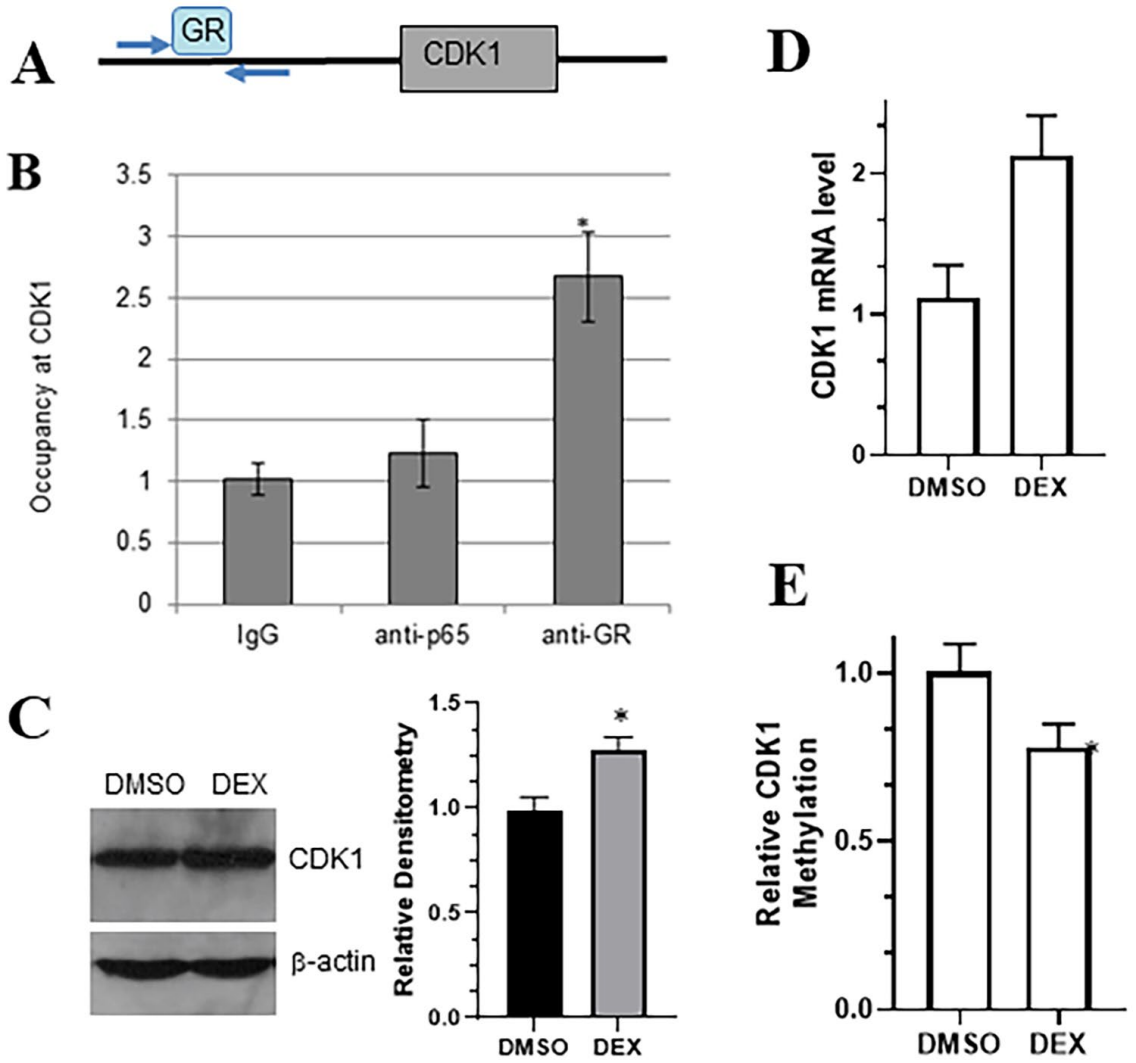
DEX-GR signaling stimulates CDK1 gene expression in T84 colon carcinoma cell line. (**A**) Schematic presentation of ChIP assay with relative position of used primers. (**B**) ChIP was performed to determine occupancy of GR at *CDK1*. The bars are indicative of the average fold enrichment, representing the standard deviation from three independent experiments. The average of fold enrichment with IgG was defined as 1. *p < 0.01, as determined by one-way ANOVA with Dunnett’s test. (**C**) Representative image from western analysis of whole cell lysates from T84 colon carcinoma cell line transfected with DEX at 1 μM for 24 hr. Densitometric analysis of CDK band from the representative western band was done, respective to the control β-actin band, with the value of CDK band under control DMSO conditions set as 1. *p < 0.01 (**D**) mRNA levels of CDK1 were assessed by RT-qPCR. *p < 0.01. (**E**) Methylation was quantitated in control (DMSO) vs. DEX-treated T84 cells. Methylation in control cells was set to 1 and relative methylation of CDK1 in treatment group was calculated. *p < 0.01.

Cancer proliferation and invasion is predominantly caused by dysregulation of CDKs^15–18^. In order to evaluate whether glucocorticoid-GR signaling involves regulation through CDK1, particularly in metastatic colon cancer cells, we first used ChIP assay to determine the occupancy of GR at CDK1 gene promoter (Fig. 3A,B). p65 was used as a control. Furthermore, we found that DEX exposure of T84 cells resulted in an increase in CDK1 expression at protein (Fig. 3C) and mRNA levels (Fig. 3D). Epigenetic regulation, such as methylation, also plays a role in eventual expression of genes^19^. Therefore, we also checked for the methylation of CDK1 in T84 cells treated with control or DEX. DEX-treated cells had significantly reduced methylation and this can explain the increased expression of CDK1. These results suggest that glucocorticoid-GR signaling stimulates CDK1 expression in metastatic colon carcinoma. There seems to be epigenetic mechanisms involved as well, which need to be studied further.

### CDK1 stimulates proliferation and invasion of metastatic colon carcinoma cells

**Figure 4:**
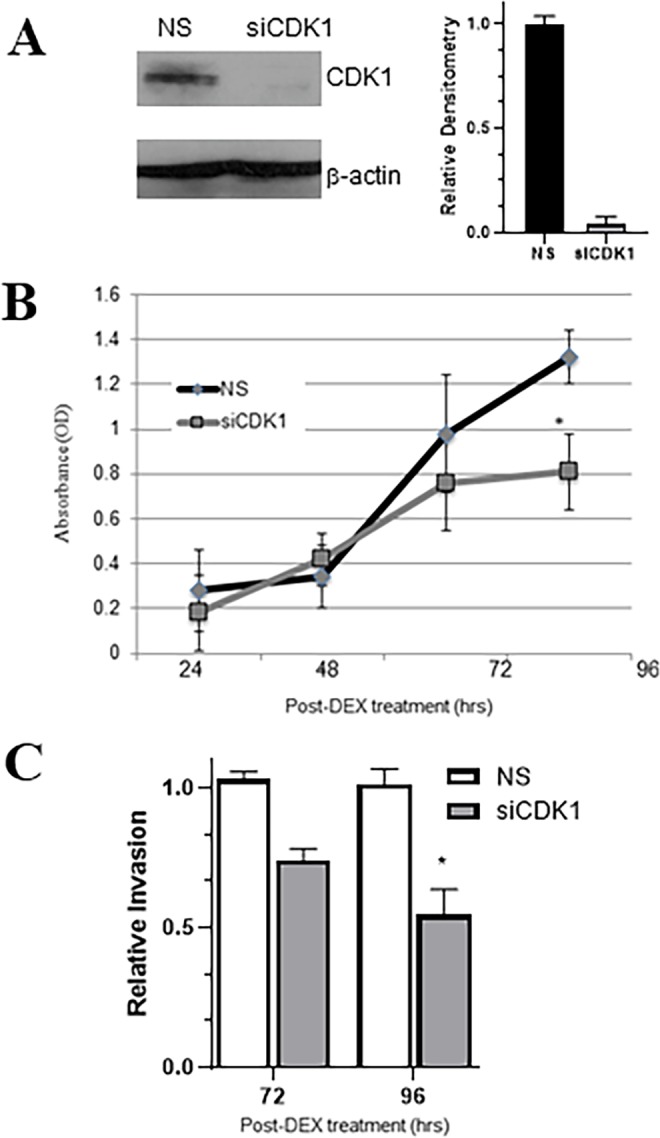
CDK1 regulates proliferation and invasion of metastatic colon carcinoma cells. Colon adenocarcinoma cell line T84 was transfected with siRNA against CDK1 as indicated for 48 hr followed by DEX treatment. (**A**) Representative image from western analysis of whole cell lysates verifying the efficacy of CDK1 silencing (n = 3 experiments). Densitometric analysis of CDK1 band from the representative western band was done, respective to the control β-actin band, with the value of CDK1 band under NS conditions set as 1. (**B**) The proliferation of colon carcinoma was analyzed by MTT proliferation and (**C**) the invasion analyzed by invasion assay (n = 3 individual experiments). Invasion of control group (DMSO) was set to 1, and relative invasion of CDK1-silenced group is reported. *p < 0.01.

Human metastatic colon cell line T84 were transiently transfected with CDK1 siRNA for 48 hr with non-specific siRNAs as the controls. The depletion of CDKI was confirmed by western blot (Fig. 4A). MTT proliferation assay showed that inhibition of CDK1 suppressed cancer cell proliferation (Fig. 4B) as well as invasion (Fig. 4C), in a time-dependent manner. These results suggest that glucocorticoid-GR signaling stimulated-proliferation and invasion is mediated by increased expression of CDK1 gene in metastatic colon carcinoma.

## Discussion

**Figure 5:**
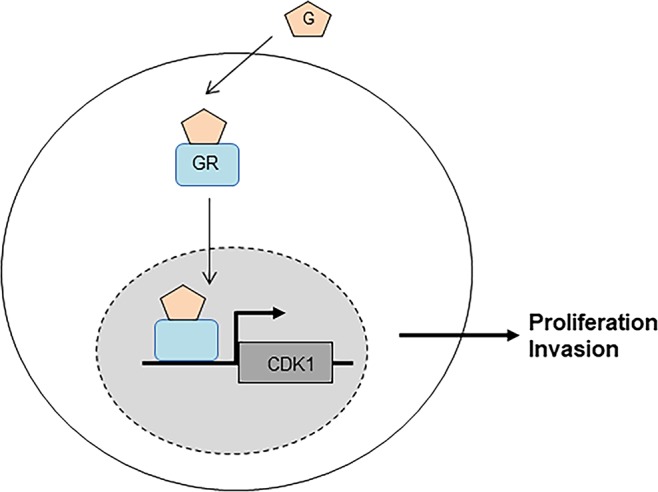
Mechanism of glucocorticoid-GR-CDK1 signaling in promoting proliferation and invasion of metastatic colon carcinoma. G, glucocorticoids; GR, glucocorticoid receptor.

CRC is one of the major human cancers that is responsible for numerous deaths through-out the world. Despite the research efforts over past several decades, there has been no major breakthrough to substantially improve the way CRC patients are treated in the hospitals. As a result, there is urgent need to doscover novel diagnostic and prognostic markers of CRC for the ultimate goal of improving the outcome of CRC patients. As a step in this direction, here we show that glucocorticoid-GR signaling plays an important role in proliferation of metastatic human colon carcinoma cells, at least in part, by inducing *CDK1* gene expression (Fig. 5).

Cancer heterogeneity poses as one of the major blockades for treatment of a variety of metastatic cancers^20^ including colon cancer^21^. It has been reported that levels of stress hormones are elevated in patients with metastatic cancer including breast cancer, compared to those with age-matched healthy counterparts or patients without metastases^22^. In addition, patients of advanced breast cancer with deregulated cortisol rhythms usually have shorter survival^23^. Very recently, a study by Obradovic *et al*. demonstrated that the increase of stress hormones during breast cancer progression leads to the activation of the glucocorticoid receptor (GR) at distant metastatic sites, increased colonization but decreased survival^24^. We found that the stress hormone pathway is an effective inducer of proliferation of metastatic colon carcinoma. Furthermore, our data have demonstrated that inhibition of GR abolished glucocorticoid-mediated upregulation of cancer cell proliferation and invasion. As a result, our results support that caution is needed when administering corticosteroids to cancer patients, as proposed^24^, and inhibition of GR may actually be helpful for chemotherapy of metastatic colon cancer.

CDKs regulate the cell cycle by interacting with cyclins. CDK1 is the principal CDK that functions to promote G2-M transition, and regulates G1 progression and the G1-S transition within the cell cycle^25,26^. Cell proliferation, one of the hallmarks of malignant tumors, is often promoted by dysregulation of CDK activity, and change in CDK expression and/or activity is frequently observed in many human cancers^27^. Other than cell-cycle regulation, CDK1 is involved in apoptosis by caspase phosphorylation including the inhibitory phosphorylation of caspase-8. To this end, Zhang and colleagues have demonstrated that targeting CDK1 is an effective strategy to enhance the efficacy of MEK inhibition in mutant *BRAF* colorectal cancer cell lines and tumor xenografts^28^, consistent with a previous report on the relevance of CDK1 as a therapeutic target in human colorectal cancers^29^. In the current study, we have shown that depletion of either GR or CDK1 halted proliferation of metastatic colon carcinoma. These results further support the high relevance of GR or CDK1 as a therapeutic target in human colorectal cancer, especially for distant metastasis.

In conclusion, we identify glucocorticoid-GR-CDK1 signaling as a novel mediator of proliferation in metastatic colorectal cancer cells. Taken together, our data support development of novel strategies to inhibit CDK1 and/or GR for the treatment of metastatic human colorectal cancers.

## Data Availability

All the data collected and analyzed for this study can be found in the submitted results (Figures).

## References

[1] Jemal A (2011). Global cancer statistics. CA Cancer J Clin.

[2] Ogata-Kawata H (2014). Circulating exosomal microRNAs as biomarkers of colon cancer. PLoS One.

[3] Chen Y (2010). Survival of metastatic colorectal cancer patients treated with chemotherapy in Alberta (1995–2004). Support Care Cancer.

[4] Brenner, H., Kloor, M. & Pox, C. P. Colorectal cancer. *Lancet* **383**, 1490–1502, doi:S0140-6736(13)61649-9 (2014). 10.1016/S0140-6736(13)61649-9 24225001

[5] Garden OJ (2006). Guidelines for resection of colorectal cancer liver metastases. Gut.

[6] Simmonds PC (2000). Palliative chemotherapy for advanced colorectal cancer: systematic review and meta-analysis. Colorectal Cancer Collaborative Group. BMJ.

[7] Simmonds PC (2006). Surgical resection of hepatic metastases from colorectal cancer: a systematic review of published studies. Br J Cancer.

[8] Holt LJ (2009). Global analysis of Cdk1 substrate phosphorylation sites provides insights into evolution. Science.

[9] Ravindran Menon D (2018). CDK1 Interacts with Sox2 and Promotes Tumor Initiation in Human Melanoma. Cancer Res.

[10] Malumbres M, Barbacid M (2009). Cell cycle, CDKs and cancer: a changing paradigm. Nat Rev Cancer.

[11] Wang XK (2016). Mono-(2-Ethylhexyl) Phthalate Promotes Pro-Labor Gene Expression in the Human Placenta. PLoS One.

[12] Li LC, Dahiya R (2002). MethPrimer: designing primers for methylation PCRs. Bioinformatics.

[13] He D (2014). MiR-371-5p facilitates pancreatic cancer cell proliferation and decreases patient survival. PLoS One.

[14] Ding L, Yu LL, Han N, Zhang BT (2017). miR-141 promotes colon cancer cell proliferation by inhibiting MAP2K4. Oncol Lett.

[15] Vermeulen K, Van Bockstaele DR, Berneman ZN (2003). The cell cycle: a review of regulation, deregulation and therapeutic targets in cancer. Cell Prolif.

[16] Casimiro MC, Crosariol M, Loro E, Li Z, Pestell RG (2012). Cyclins and cell cycle control in cancer and disease. Genes Cancer.

[17] García-Reyes Balbina, Kretz Anna-Laura, Ruff Jan-Philipp, von Karstedt Silvia, Hillenbrand Andreas, Knippschild Uwe, Henne-Bruns Doris, Lemke Johannes (2018). The Emerging Role of Cyclin-Dependent Kinases (CDKs) in Pancreatic Ductal Adenocarcinoma. International Journal of Molecular Sciences.

[18] Lee DE (2011). 6,7,4′-trihydroxyisoflavone inhibits HCT-116 human colon cancer cell proliferation by targeting CDK1 and CDK2. Carcinogenesis.

[19] Farhan Mohd, Ullah Mohammad, Faisal Mohd, Farooqi Ammad, Sabitaliyevich Uteuliyev, Biersack Bernhard, Ahmad Aamir (2019). Differential Methylation and Acetylation as the Epigenetic Basis of Resveratrol’s Anticancer Activity. Medicines.

[20] Dagogo-Jack I, Shaw AT (2018). Tumour heterogeneity and resistance to cancer therapies. Nat Rev Clin Oncol.

[21] Blank A, Roberts DE, Dawson H, Zlobec I, Lugli A (2018). Tumor Heterogeneity in Primary Colorectal Cancer and Corresponding Metastases. Does the Apple Fall Far From the Tree?. Front Med (Lausanne).

[22] van der Pompe G, Antoni MH, Heijnen CJ (1996). Elevated basal cortisol levels and attenuated ACTH and cortisol responses to a behavioral challenge in women with metastatic breast cancer. Psychoneuroendocrinology.

[23] Sephton SE, Sapolsky RM, Kraemer HC, Spiegel D (2000). Diurnal cortisol rhythm as a predictor of breast cancer survival. J Natl Cancer Inst.

[24] Obradovic MMS (2019). Glucocorticoids promote breast cancer metastasis. Nature.

[25] Enserink JM, Kolodner RD (2010). An overview of Cdk1-controlled targets and processes. Cell Div.

[26] Otto T, Sicinski P (2017). Cell cycle proteins as promising targets in cancer therapy. Nat Rev Cancer.

[27] Ortega S, Malumbres M, Barbacid M (2002). Cyclin D-dependent kinases, INK4 inhibitors and cancer. Biochim Biophys Acta.

[28] Zhang P (2018). Targeting CDK1 and MEK/ERK Overcomes Apoptotic Resistance in BRAF-Mutant Human Colorectal Cancer. Mol Cancer Res.

[29] Atlas N, Cancer Genome (2012). Comprehensive molecular characterization of human colon and rectal cancer. Nature.

